# Bioremediation of a Complex Industrial Effluent by Biosorbents Derived from Freshwater Macroalgae

**DOI:** 10.1371/journal.pone.0094706

**Published:** 2014-06-11

**Authors:** Joel T. Kidgell, Rocky de Nys, Yi Hu, Nicholas A. Paul, David A. Roberts

**Affiliations:** 1 MACRO - The Centre for Macroalgal Resources and Biotechnology, and School of Marine and Tropical Biology, James Cook University, Townsville, Queensland, Australia; 2 Advanced Analytical Centre, James Cook University, Townsville, Queensland, Australia; University of New South Wales, Australia

## Abstract

Biosorption with macroalgae is a promising technology for the bioremediation of industrial effluents. However, the vast majority of research has been conducted on simple mock effluents with little data available on the performance of biosorbents in complex effluents. Here we evaluate the efficacy of dried biomass, biochar, and Fe-treated biomass and biochar to remediate 21 elements from a real-world industrial effluent from a coal-fired power station. The biosorbents were produced from the freshwater macroalga *Oedogonium* sp. (Chlorophyta) that is native to the industrial site from which the effluent was sourced, and which has been intensively cultivated to provide a feed stock for biosorbents. The effect of pH and exposure time on sorption was also assessed. These biosorbents showed specificity for different suites of elements, primarily differentiated by ionic charge. Overall, biochar and Fe-biochar were more successful biosorbents than their biomass counterparts. Fe-biochar adsorbed metalloids (As, Mo, and Se) at rates independent of effluent pH, while untreated biochar removed metals (Al, Cd, Ni and Zn) at rates dependent on pH. This study demonstrates that the biomass of *Oedogonium* is an effective substrate for the production of biosorbents to remediate both metals and metalloids from a complex industrial effluent.

## Introduction

Mining, mineral processing and energy generation produce large quantities of contaminated effluent. For example, coal-fired power stations produce complex effluents containing dissolved elements from the flushing of ash from the flue and furnace [1]. The resulting effluent contains elements at concentrations of potential environmental concern, such as Al, As, B, Cd, Mo, Se, Sr, V, and Zn, and extensive treatment is required before the effluent can be discharged [1], [2]. As the cost and operational conditions of treatment options can be prohibitive [1], [3], the effluent is often retained in large storages known as Ash Dams (AD). However, despite the apparent confinement of these water bodies, AD remain a significant source of toxic elements to local organisms [4]. Consequently, there is a need for a cost effective, sustainable and comprehensive approach to the remediation of complex industrial effluents.

Biosorption with biomass is an alternative to existing waste water treatment technologies with promising results at the laboratory scale [5]. Biosorption exploits the ability of dead or denatured biomass, such as dried macroalgae, to passively bind ions from aqueous solutions [6], [7]. Dried macroalgae are particularly effective biosorbents due to the high abundance of functional groups which have a strong affinity for dissolved cationic metals despite also having relatively high concentrations of these same metals in the biomass [8]. Many functional groups can be involved in biosorption and this can vary according to taxonomic groupings. For example, in brown algae the carboxylic groups of alginates are typically dominant in biosorption processes, while some freshwater green algae, such as *Oedogonium*, have cellulosic cell walls that resemble those of higher plants [9], [10]. These functional groups can passively bind dissolved metals through various processes, including passive electrostatic attraction, ion exchange with “light” metal ions (Ca^2+^, Na^+^, K^+^ and Mg^2+^), or complexation processes [10].

Macroalgae (and other biosorbents such as activated carbon) only have an affinity for dissolved cations and are relatively ineffective at treating oxyanions, such as selenate (SeO_4_^2−^) that are common constituents of effluents [11]. However, dried macroalgae can be manipulated to improve its affinity for specific contaminants. Biomass can be converted to carbon-rich biochar through slow pyrolysis, resulting in a product with similar properties to activated carbon [12]. Additionally, biomass and biochar can be pre-treated with an iron (Fe) solution to improve the adsorption of anionic metalloids, including SeO_4_^2−^ [13]. Deposition of Fe onto the surface of either dried biomass or biochar provides a positive charge, promoting the formation of inner-sphere complexes between oxyanionic metalloids and Fe-treated biosorbents, where there would otherwise be no natural affinity for sorption [14]–[16].

Despite the promise of macroalgae as a biosorbent, industrial application has been limited. One key factor limiting the application of biosorption is the lack of a sustainable and sufficient source of biomass [6], [17]. Wild harvests of biomass to support biosorption are simply not sustainable when one considers the volumes required [6], and commercially cultivated seaweeds have existing applications in other markets [17]. However, in recent work we have shown that native species of macroalgae can be cultured to provide sustainable biomass for bioremediation [17], [18]. This cultivated biomass represents a sustainable source of biomass, but little research has considered the efficacy of cultivated biomass in biosorption applications.

An additional limitation of existing biosorption research is that it has focused on simple synthetic effluents. These studies often focus on the kinetics of sorption and the mechanisms of uptake of select elements under idealized conditions. In contrast, real-world industrial effluents are complex, involving multiple interacting and competing contaminants that occur in a variety of speciation and oxidation states, which are influenced by environmental conditions [2], [8]. Biosorption research that has been conducted in multi-element systems has shown that non-target elements can interfere with [19], [20] or competitively exclude [21] biosorption of target elements. Consequently, in multi-element systems the capacity of a biosorbent for individual elements typically decreases in comparison to results obtained in idealized single-element effluents [22]. Macroalgal biosorbents have not yet been proven to be an effective means of treating complex effluents with multiple co-existing contaminants [7] and it is rare for studies to consider systems with more than three elements [8]. In fact, very little is known about the performance of biosorbents of any type in multi-elemental systems, or the effects that physical parameters such as pH and exposure time have in these scenarios.

Here we address key constraints to the industrial application of algal-based biosorption by assessing the efficacy of a macroalgal biosorbent for use in a real-world complex industrial effluent. We collect a native isolate of the cosmopolitan freshwater macroalgal genus *Oedogonium* (Link ex Hurn, 1900) from the AD of a coal-fired power station and cultivate it in intensive production systems as a means of providing sustainable biomass for biosorption. Specifically, we test *Oedogonium* dried biomass, derived biochar, Fe-treated biomass and Fe-treated biochar as biosorbents for 21 metals and metalloids in an effluent taken from coal-fired power production under a range of pH conditions and exposure times. These results will establish the potential of biosorption for the remediation of complex industrial effluents using purposely cultivated biomass.

## Materials and Methods

### Industrial effluent

This study targeted Ash Dam Water (ADW) from Tarong coal-fired power station in south-east Queensland, Australia (26.76°S, 151.92°E). Tarong is one of Queensland's largest power stations with a generation capacity of 1400 MW, and a 46,000 ML AD storing contaminated waste water. ADW was sourced directly from the AD and transported to James Cook University (JCU), Townsville in 1000 L Intermediate Bulk Containers (IBCs) in November 2012. The ADW was then stored at ambient temperature in 12,000 L storage tanks until use. The effluent was collected and transported to JCU by Stanwell Energy Corporation.

### Algal biosorbent production & preparation

*Oedogonium* sp. (Genbank: KF606974) [23] hereafter *Oedogonium*, was used as the source biomass for the production of biosorbents (see below). *Oedogonium* is a native filamentous, freshwater green alga in the Tarong AD [18]. *Oedogonium* samples were initially collected from the Tarong AD in October 2012 but could not be identified to species using taxonomic keys based on morphological characteristics [24]. The species was therefore assessed using molecular techniques, arguably the most accurate means to identify cryptic species, and this isolate has been assigned the Genbank accession number KC606974 with no current matches for this species in the database [23].

After collection from Tarong AD, *Oedogonium* was cultivated in Manutec f/2 algal growth media in 2500 L tanks during the austral summer months (January – March) in the aquaculture facility at JCU (19.33°S, 146.76°E). Prior to experiments, 2 kg of algae was harvested from the tanks and oven dried to a constant mass at 60°C for 48 hours (h). Subsequently, 1 kg of the dried *Oedogonium* biomass was converted into biochar by slow pyrolysis under conditions previously developed for macroalgae [12]. Briefly, *Oedogonium* was suspended within a muffle furnace (Labec CEMLS-1200) and continuously purged with N_2_ (BOC) gas at 4.0 L min^−1^ while being heated to a hold temperature of 450°C for 1 h. Additionally, a sub-sample of both the dried biomass and biochar were also treated with a 5% Fe solution, prepared by diluting FeCl_3_ (Sigma Aldrich 45% w/v) in deionized (DI) water (Millipore Direct-Q3), to become Fe-loaded biosorbents. Dried biomass and biochar were exposed to separate Fe solutions at a density of 25 g L^−1^ for 24 h on a shaker plate (100 rpm) at 20°C, then filtered from the solutions and rinsed three times with DI water at a rate of 20 ml g^−1^, then dried at 60°C for 48 h.

### Biosorption experiments

Biosorption experiments were conducted to quantify the rate and composition of metal and metalloid adsorption from ADW by *Oedogonium* dried biomass, biochar, Fe-treated dried biomass and Fe-treated biochar at three alternate initial pH levels (2.5, 4, 7.1 see below). Filtered (0.45 µm sterile Starstedt syringe filters) samples of ADW were analyzed prior to experimental treatments to serve as a benchmark for initial conditions.

Two solutions of pH-manipulated ADW were produced with 1 M HCl (pH 2.5 and 4, Sigma Aldrich TraceSelect Ultra), while a third remained at the native pH of the ADW, 7.07±0.01. The experiment was fully factorial in design, with independent samples being destructively sampled at each time point. Each of the treatments consisted of a plastic beaker with 60 ml of ADW and 0.6 g of biomass, biochar or the Fe-treated derivatives (10 g L^−1^ of biosorbent). The beakers were shaken (100 rpm at 20°C) in incubator shaker cabinets. At the end of the allocated exposure time (0:15, 0:30, 1:00, 4:00, 24:00, or 168:00 hours) the samples were removed from the cabinets and filtered with 75 µm nylon filter paper. The solution was then filtered to 0.45 µm using a glass fiber filter and syringe, then analysed as described below. Samples containing no biosorbent were processed in the same manner to serve as negative controls to quantify losses of elements to experimental glassware and filtration. The experiment was replicated three times. All plastic and glassware was acid washed in a 5% HNO_3_ (Sigma Aldrich) bath for 48 h, then rinsed in DI water prior to use.

### Elemental analysis

The concentrations of Al, As, Ba, Cd, Co, Cr, Cu, Fe, Mn, Mo, Ni, Pb, Se, Sr, V and Zn were measured with a Bruker 820-MS Inductively Coupled Plasma Mass Spectrometer (ICP-MS), and Ca, K, Mg and Na with a Varian Liberty series II Inductively Coupled Plasma Optical Emissions Spectrometer (ICP-OES). An external calibration strategy was used for both instruments, where a standard solution of 0.45 µm filtered ADW was used as the vector to calculate the concentration of elements. Collisional Reaction Interface (CRI) was used for As (H_2_) and V (He), while ^82^Se isotope was used for Se quantification, to eliminate polyatomic interferences for these elements. A 1% HCl solution was spiked with 1 ppb As, Se and V and measured three times for quality control; recovery between 98.5 and 110% indicated no significant interferences. All analyses were conducted at the Advanced Analytical Centre at JCU, Townsville.

### Data analysis

Multivariate patterns in biosorption were visualized using Principal Components Analysis (PCA) from a correlation matrix with some elements log-transformed to create a normal distribution [25]. Univariate analysis took the form of three-way fully-factorial Analysis of Variance (ANOVA), with the factors biosorbent (fixed), pH (fixed) and time (random). Data were examined for normality and homogeneity of variance using normal-probability plot of raw residuals and predicted-residual scatter plot, and were transformed as necessary to meet assumptions [26]. Both the PCA and ANOVA test were conducted in Statistica for Windows (Ver. 10, C. Statsoft Inc. 1984–2011).

## Results

### Characteristics of ADW

Twelve (Al, As, B, Cd, Cr, Cu, Pb, Mn, Mo, Ni, Se and Zn) of the 21 elements measured in the ADW have trigger levels established by the Australian and New Zealand Environmental Conservation Council (ANZECC) [27]. Of these twelve elements, eleven (Al, As, B, Cd, Cr, Cu, Pb, Mo, Ni, Se and Zn) were in excess of the trigger values (Table 1). Given that these elements have quantifiable remediation goals they are the focus of the following results section.

**Table 1:** The ANZECC trigger level and initial concentration for each element investigated, in addition to the lowest final concentration and the biosorbent, time and pH conditions responsible.

Element	ANZECC Trigger (µg L^−1^)	Initial Concentration [µg L^−1^ (± SE)]		Final Concentration [µg L^−1^ (± SE)]		Best Biosorbent	Fastest Time	Best pH
**Aluminium**	**55**	**144**	**(35)**	**35**	**(28)**	**Biochar**	**0:30**	**≥4**
**Arsenic**	**13**	**43**	**(5.5)**	**9**	**(1.0)**	**Fe-Biochar**	**24**	**NA**
Boron	370	7475	(893)	5767	(462)	Fe-Biochar	0:15	4
Cadmium	0.2	2.3	(0.2)	0.9	(0.2)	Biochar	0:30	≥4
Chromium	1	5.2	(3.3)	2.6	(2.1)	Biochar	0:15	NA
**Copper**	**1.4**	**1.9**	**(0.9)**	**1.2**	**(0.9)**	**Biochar**	**0:30**	**NA**
Lead	3.4	0.3	(0.1)	0.03	(<0.01)	Biochar	0:30	8
Manganese	1900	3	(0.8)	-		**-**	**-**	**-**
**Molybdenum**	**34**	**1437**	**(127)**	**28**	**(5.9)**	**Fe-Biochar**	**168**	**NA**
**Nickel**	**11**	**53**	**(7.3)**	**11**	**(2.7)**	**Biochar**	**24**	**≥4**
Selenium	11	82	(3.9)	21	(2.6)	Fe-Biochar	0:30	NA
Selenium	11	82	(3.9)	13	(1.8)	Biomass	168	4
**Zinc**	**8**	**64**	**(11)**	**5**	**(2.4)**	**Biochar**	**0:30**	**≥4**
Barium	-	108	(2.3)	90	(4.2)	Biochar	168	NA
Calcium	-	330500	(1528)	293000	(3464)	Biochar	168	≥4
Cobalt	-	0.6	(0.1)	0.3	(0.1)	Biochar	0:30	≥4
Iron	-	1372	(360)	671	(211)	Biochar	1	NA
Magnesium	-	93700	(302)	-		-	-	-
Potassium	-	30022	(11416)	-		-	-	-
Sodium	-	446000	(2363)	396667	(14170)	Biochar	4	≥4
Strontium	-	1648	(275)	-		-	-	-
Vanadium	-	1098	(102)	149	(31)	Biomass	168	≥4

Elements which were reduced below ANZECC trigger level are in bold.

“NA” indicates that the lowest concentration was not significantly different between pH conditions.

“-” indicates element did not change or only increased in concentration.

### Biochar, biomass and ANZECC metals

**Figure 1:**
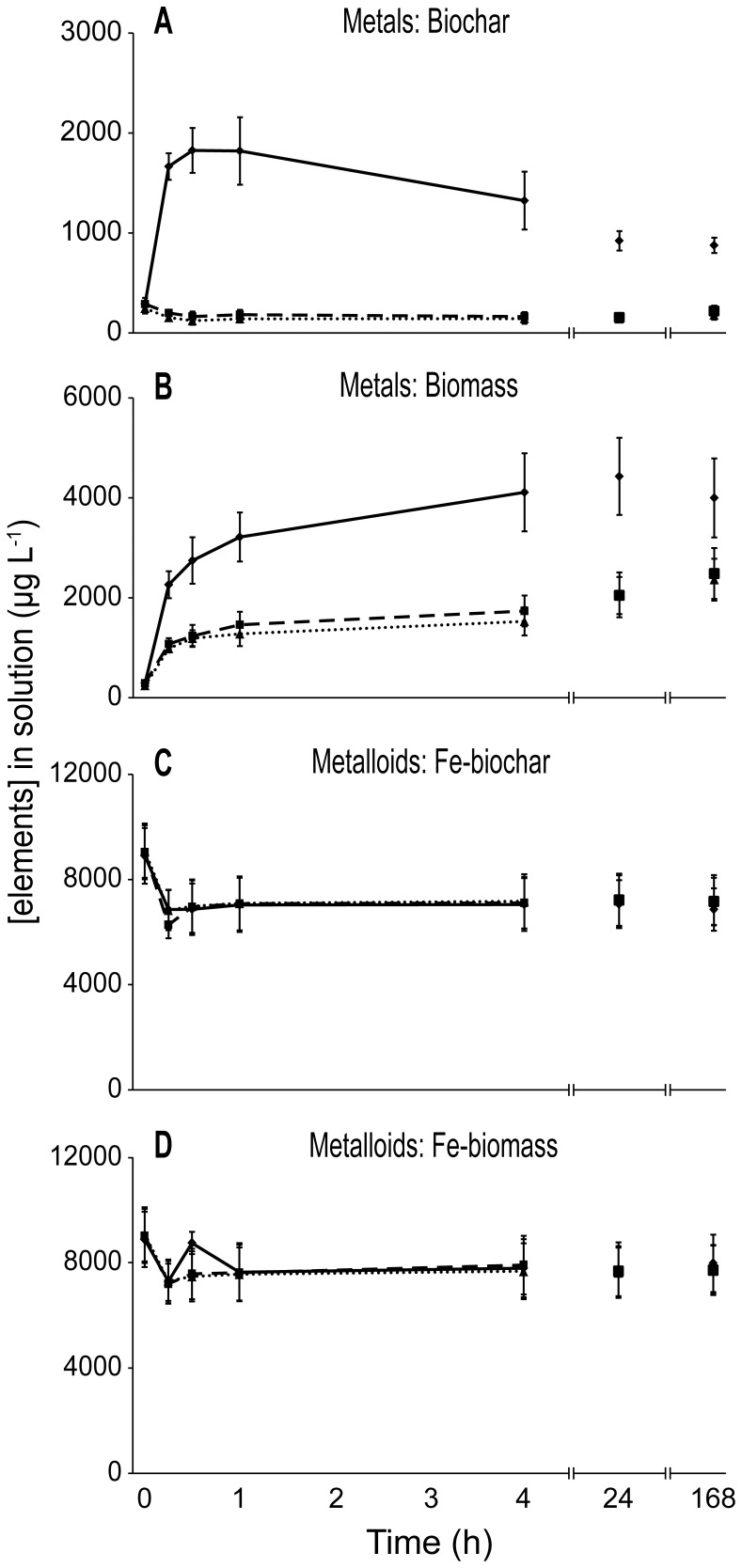
Total respective biosorption of ANZECC metals by biochar and biomass, and metalloids by Fe-treated biosorbents. Biosorption of metals (Al, Cd, Cr, Cu, Pb, Mn, Ni, and Zn) by (a) biochar and (b) biomass and biosorption of metalloids (As, B, Mo and Se) by (c) Fe-biochar and (d) Fe-biomass. Initial pH of 2.5, 4 and un-manipulated (7.1) are shown by solid, dashed and dotted lines, respectively. Error bars show standard errors

**Figure 2:**
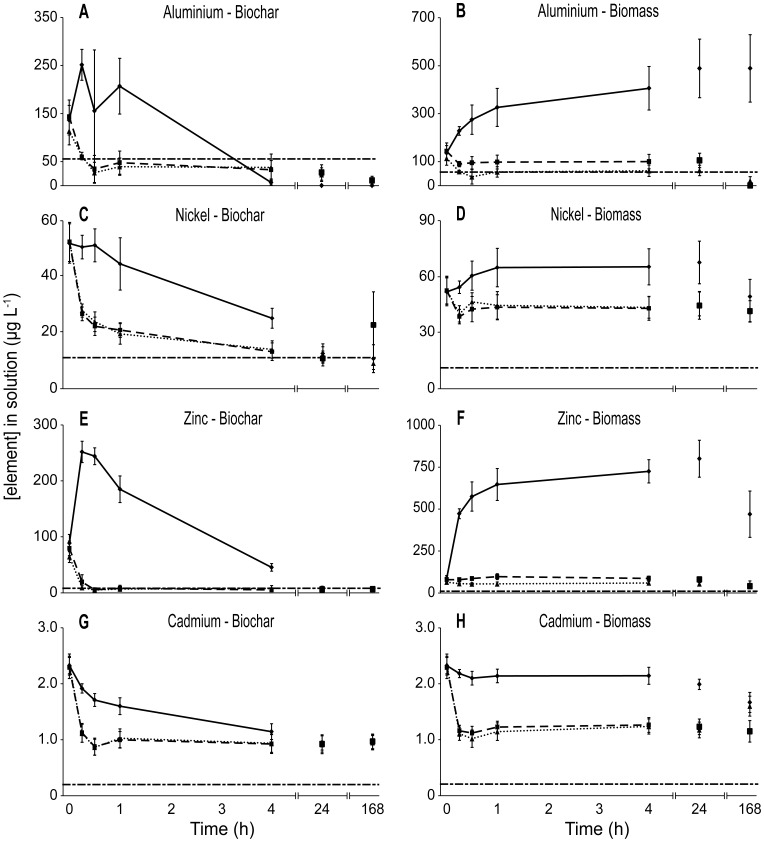
Biosorption of ANZECC metals (Al, Ni, Cd, and Zn) when exposed to biochar and biomass. Initial pH of 2.5, 4 and un-manipulated (7.1) are shown by solid, dashed and dotted lines, respectively. Error bars show standard errors. Horizontal dashed line indicates the respective ANZECC trigger concentration for each element.

Biochar was the most effective biosorbent, removing a broad suite of metals (Mn, Al, Cr, Cu, Cd, Ni, Pb and Zn). from solution (Figures 1 and 2). The PCA shows that effluent treated by biochar clustered along the positive PC1 axis, being characterized by lower concentrations of metals than the effluents treated by the remaining biosorbents (Figure S1). There was, however, a significant effect of pH on the biosorption of most metals by biochar (“Biosorbent x pH” Table S1). At high initial pH (4.0 & 7.1) the raw biochar rapidly adsorbed metals from solution but at low initial pH (2.5), leached metals into solution (Figure 1a).

Of the metals included in the ANZECC guidelines, Al, Ni and Zn were most effectively removed from solution by biochar, and each of these had a pH-dependent response in the rate of biosorption (Figure 2a, c, e; Table S1). All three of these metals were reduced below their respective ANZECC trigger levels by biochar at high initial pH, in the case of Al and Zn within 30 min (Table 1; Figure 2a, c, e). When pH was initially low (2.5), the concentration of Al and Zn increased in solution in the first 15 min, then over the next four hours adsorbed onto the biochar to finally reach levels below the limits of detection (Figure 2a, e). While Cd was also adsorbed by biochar at varying rates under different initial pH conditions (2.5, 4.0 and 7.1), it was not reduced to below the trigger level (Figure 2g; Table 1).

The response of metals to biomass varied greatly and, as with biochar, often in a pH-mediated fashion (Figure 1b). Overall, there was an increase in element concentrations in ADW treated with biomass (Figure 1b) which is supported by effluent treated by biomass being broadly distributed around the centroid in the PCA, demonstrating it was a relatively ineffective biosorbent (Figure S1). Al was the only metal reduced below its respective trigger level when exposed to biomass, and this only occurred at high initial pH (4 and 7.1) (Figure 2b). In contrast, at an initial pH of 2.5 the concentration of Al increased substantially and continuously for the entire exposure duration (Figure 2b). Again, Zn displayed a similar pattern (Figure 2f). When exposed to biomass, Ni and Cd both displayed a similar pattern of initial decrease in concentration at high pH (4.0 and 7.1) followed by no significant change for the remaining duration of exposure, however, in low initial pH (2.5) both Ni and Cd did not differ from the initial concentration (Figure 2d, h; Table S1). Mn displayed substantial pH-mediated leaching when exposed to both biochar and biomass (Figure. S3g, h).

### Fe-biochar, Fe-biomass and ANZECC metalloids

Fe-biochar was an effective adsorbent of As, Mo and Se, and initial pH had no impact on the rate or extent of adsorption of these elements (Figure 1c, Table S1). The net concentration of all ANZECC oxyanionic metalloids (As, B, Mo, Se) decreased by 2700 µg L^−1^ (30%) within the first 15 min of exposure to Fe-biochar when the initial pH was 4 or 7.1 (Figure 1c). This can be visualized in the PCA, in which ADW treated with Fe-biochar clusters along the positive PC2 axis in the PCA, demonstrating the effluent treated with Fe-biochar tended to have lower concentrations of As, Se and Mo than the remaining treatments (Figure S1).

**Figure 3:**
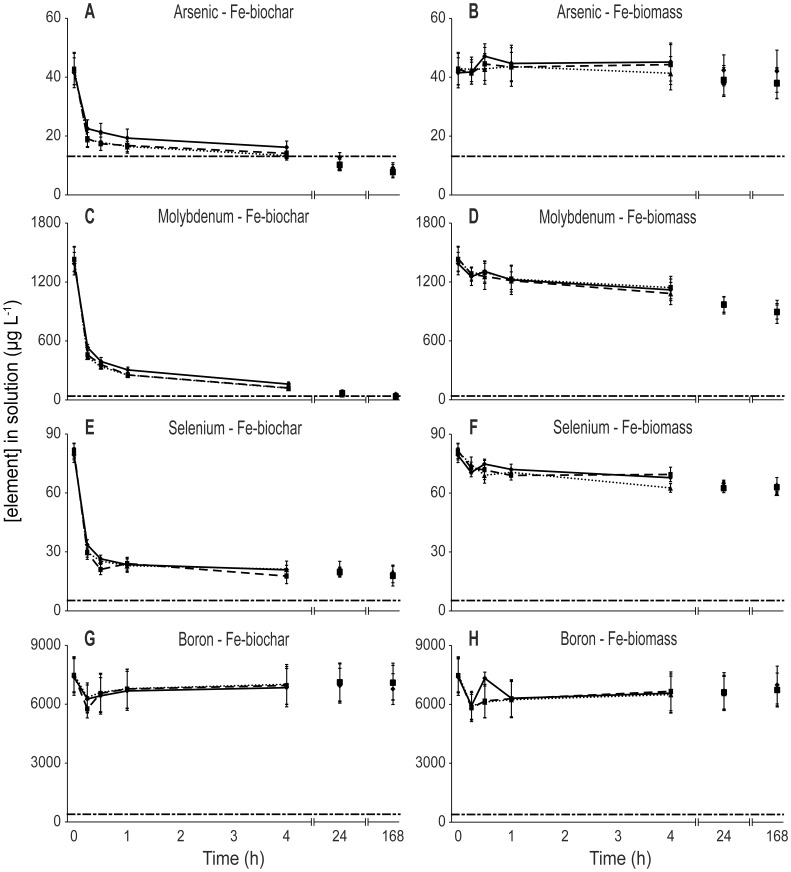
Biosorption of ANZECC metalloids (As, B, Mo and Se) when exposed to Fe-biochar and Fe-biomass. Initial pH of 2.5, 4 and un-manipulated (7.1) are shown by solid, dashed and dotted lines, respectively. Error bars show standard errors. Horizontal dashed line indicates the respective ANZECC trigger concentration for each element.

The concentrations of As, Mo and Se all dropped significantly lower with Fe-biochar than for any other biosorbent. As and Mo were adsorbed by Fe-biochar to below their respective trigger levels for all initial pH conditions (Table 1; Figure 3a, c). Se was substantially reduced within the first 15 mins by Fe-biochar but not to the point of the AZNECC trigger level (Figure 3e; Table 1). As and Mo followed the characteristic pattern of rapid initial adsorption within the first 15 mins and continued decline at a slower rate for the remaining exposure (Figure 3a, c). Initial concentrations of B were 20 times in excess of the trigger level and despite a drop in concentration of approximately 20% when exposed to Fe-biochar at an initial pH of 4 (Table 1), the concentration of B did not approach the trigger level for any of the treatments (Figure 3g, h).

Fe-biomass behaved in a similar manner to Fe-biochar, albeit not as successfully. There was an initial reduction of 1700 µg L^−1^ (18%) of ANZECC metalloids in the first 15 min of exposure to Fe-biomass (Figure 1c). Interestingly, untreated biomass at low pH (2.5) showed a similar total effectiveness with metalloids as Fe-biochar, with an initial decrease in metalloid concentration of 2400 µg L^−1^ (28%) in the first 15 min (Figure. S2d).Mo and Se were slightly reduced in ADW when exposed to Fe-biomass (Figure 3d, f; Table S1), however, these were the only elements to do so.

## Discussion

This study demonstrates that the biomass of *Oedogonium* is an effective substrate for the production of biosorbents to remediate both metals and metalloids from a complex industrial effluent. Conversion of biomass to biochar through slow pyrolysis, and Fe-treatment of this biochar, produces biosorbents that effectively bind metals and metalloids respectively. The affinity of each biosorbent for different constituents of an extremely complex waste water effluent is clearly demonstrated where biochar binds metals from solution at a rate that is affected by pH, while Fe-biochar consistently binds metalloids from solution in a manner that is unaffected by pH. Our results therefore highlight the complexities of biosorption that are only apparent in experiments conducted on real-world industrial effluents. No single biosorbent was effective at holistically treating the range of elements in the complex ADW and so biosorption strategies for real-world effluents may require multiple stages of treatment.

The greatest change in metal and metalloid concentration within the ADW occurred in the first hour of exposure. Rapid initial sorption of metals and metalloids is commonly reported [28]–[33]. The effect of pH was pronounced for the untreated biosorbents, biomass and biochar, with biosorption patterns at low initial pH (2.5) often differing to those at higher initial pH (4.0 and 7.1). The effect of pH for Fe-biomass and Fe-biochar was, however, negligible. The pH-independent sorption of metalloids by Fe-treated biosorbents may be due to the formation of inner-sphere complexes [14], which are largely unaffected by ionic strength and act without the restrictions of electrostatic attraction, allowing bond formation irrespective of net biosorbent charge [34].

The suites of elements targeted by the most successful biosorbents, Fe-biochar and biochar, were distinct and complementary. Fe-biochar removed the oxyanionic metalloids As, Mo and Se, with As and Mo being reduced to below their respective ANZECC trigger levels, which is particularly notable for Mo as the concentration was initially 40 times in excess of the trigger level. While the ability of Iron Based Sorbents (IBS) to remediate anions has been established [11], [12], [13], [35], we have shown that the remediation of these metalloids in a complex effluent comes at the expense of substantial leaching of metals back into solution. Conversely, biochar was able to remove a suite of metals from solution and did not leach any ANZECC elements into solution at high pH. The ability to simultaneously remove multiple metals from solution makes biochar a very successful biosorbent in a multi-elemental context, and offers the potential to combine biochar and Fe-biochar in sequential treatment strategies to sequester both metals and metalloids from complex effluents.

For biochar, there was significant variation in metal sorption with half of the ANZECC metals (Al, Cu, Ni & Zn) being remediated to below their respective trigger levels, while metals such as Mn and K leached off the biochar and into solution. Ionic affinity for biosorbents is not fully understood, however, the ionic radius and electronegativity of a metal may have a significant effect [36]–[38]. For example, cation adsorption onto freeze-dried fungus *Rhizopus arrhizus* may be related to the electronegativity and ionic radius of each ion, otherwise known as the Covalent Index, which suggests Mn^2+^ has a very low sorption affinity [39], [40]. Consequently, it is possible that during this study ion exchange is occurring involving the release of Mn, with its relatively low affinity and high abundance on *Oedogonium*, in exchange for metals of higher affinity such as Zn, Pb or Cu [36].

Metals and metalloids behaved differently when exposed to biomass and biochar under low initial pH conditions. When exposed to biomass and biochar at an initial pH of 2.5, several metals (Al, Cd, Mn, Ni, Zn) had higher concentrations in solution than the higher pH (4 & 7.1) treatments. As described earlier, the biomass was initially sourced from Tarong Ash Dam and cultivated in f/2 media. Consequently, the resulting biomass contained elements from f/2 media that are required for growth and some of these elements leached when the dried biomass was returned to water at low pH (particularly Cu, Mo, Mn, Zn and Fe). Our finding that some elements leached from biomass at low pH further highlights the importance of measuring a broad suite of analytes in biosorption experiments to uncover unexpected interactions between target and non-target elements. Conversely, metalloids (Se, Mo and V) had lower concentrations at low initial pH. There are several possible explanations for this pH-mediated response. Firstly, the increased metal concentration is a result of increased availability of free-ions at lower pH [41], [42]. Second, the metals could be competitively excluded from the biosorption sites by the increased number of protons at lower pH [43], [44]. Third, the lowering of the pH below the isoelectric point of the biosorbent resulted in a net charge reversal and therefore enhanced the adsorption of metalloids while limiting the adsorption of metals [45]–[47]. In reality the pH-dependent adsorption of ions onto biosorbents is probably due to a combination of factors [6], [7]. Overall however, lowering the pH to 2.5 in this study had no benefit to the removal of ions as elements were most successfully removed from the effluent at an un-manipulated initial pH of 7.1.

Interestingly, when the biomass was converted to biochar, the metal leaching at low pH was reduced by more than 50%. While the behavior of complex feed stocks during slow pyrolysis is relatively poorly understood, it is known that biochar produced from element-rich biomass typically has a lower exchangeable fraction of metals than the feed stock. Some elements that are constituents of biomass are volatile and may not report to the biochar fraction during slow pyrolysis. Furthermore, converting biomass to biochar changes the speciation of bound metals, rendering them less liable to dissociation [48]. This is clearly supported by the significantly lower leaching of metals from biochar at low pH in our study. Clearly, therefore, biomass cultivated using f/2 media – or any similar growth media – can be considered an appropriate feedstock for biosorption despite containing elements that are also targets for bioremediation, and this biomass is most effective when converted to biochar and used at an unmodified pH.

In an overall sense, there is a developing dichotomy in the study of biosorption of metals and metalloids. The majority of research to date has focused on the kinetics and mechanisms of biosorption in synthetic effluents, which are in essence abstract and simplified conditions. While these studies are important in understanding the processes involved in biosorption, they lack the authenticity of complex effluents in which biosorption is to be applied [5]–[7]. Our results clearly demonstrate that macroalgae are a versatile feedstock for biosorbents, as *Oedogonium* biomass was able to be converted to biochar, Fe-biomass and Fe-biochar, each of which displayed differential affinity for metals and metalloids. Determination of whether the metal leaching that occurred for Fe-biochar (during the removal of the problematic metalloids) is an acceptable outcome or if the metals could be remediated using another process or biosorbent such as a sequential treatment, requires further investigation. A sequential approach in which alternative macroalgal-derived biosorbents are used in sequence on the same effluent solution, each targeting a specific suite of elements in the effluent, may result in a more comprehensive treatment. Regardless, our results highlight the critical importance of research that evaluates biosorbent performance in industrial effluents to fully understand the potential and viability of algal-based biosorption as a water treatment technology.

### Conclusions

In conclusion we have not only demonstrated that the macroalga *Oedogonium* is an effective biosorbent in a complex industrial effluent, but we have done so with a macroalga that can be produced on-site at industrial facilities [18]. The biomass used in this study was cultivated at large scale in f/2 media to provide a rapidly growing source of biomass for waste water treatment in industry. To achieve this rapid growth, some elements must be added as part of any standard algal growth media, but these elements are minor components of the biomass relative to the positive effects of biosorption. The intensive cultivation of the biomass delivers the productivities required to support scaled biosorption which circumvents a critical barrier to application of biosorption. Furthermore, the on-site production of a native macroalga negates one of the most problematic components in the use of algal-based biosorbents, the source and transport of the biomass [6]. This offers a new paradigm in sustainable waste water treatment, where biomass for bioremediation is produced on-site at industrial facilities while delivering carbon capture. Through this strategic integration of industries, algal-based biosorption will have much greater prospects for industrial application.

## Supporting Information

Figure S1 Principal Components Analysis of solution concentration for 12 ANZECC elements. (A) PCA and (B) factor loadings for 12 elements include all biosorbent, time periods (excluding t0) and pH conditions, grouped by biosorbent. Vectors (factor loadings) indicate the direction and magnitude of correlation between a specific element and the biosorbent which resulted in the lowest respective concentration.
(DOCX)

Figure S2 The total respective biosorption of metals (Al, Cd, Cr, Cu, Pb, Mn, Ni, and Zn) by (a) Fe-biomass and (b) Fe-biochar and total respective biosorption of metalloids (As, B, Mo and Se) by (c) biochar and (d) biomass. Initial pH of 2.5, 4 and un-manipulated (7.1) are shown by solid, dashed and dotted lines, respectively. Error bars show standard errors.
(DOCX)

Figure S3 The biosorption of ANZECC metals (Pb, Cr, Cu, and Mn) when exposed to biochar and biomass. Initial pH of 2.5, 4 and un-manipulated (7.1) are shown by solid, dashed and dotted lines, respectively. Error bars show standard errors. Horizontal dashed line indicates the respective ANZECC trigger concentration for each element.
(DOCX)

Table S1 Three factor factorial Analysis of Variance tests run on each of the 12 ANZECC elements. Factorial analysis of variance tests were run on elemental concentration under the factors of Biosorbent, pH (Fixed) and Time (Random). Type III sum of squares was used. All tests met the assumption of homogeneity of variance, normality of residuals and independence. Transformation of the data were required for some elements, the transformation applied is listed next to the title. Factors in bold indicate significance under alpha of 0.05.
(DOCX)

## References

[1] Volesky B (2001) Detoxification of metal-bearing effluents: Biosorption for the next century. Hydrometallurgy 59: 203–216.

[2] Jankowski J, Ward CR, French D, Groves S (2006) Mobility of trace elements from selected Australian fly ashes and its potential impact on aquatic ecosystems. Fuel 85: 243–256.

[3] Frankenberger WT Jr, Amrhein C, Fan TWM, Flaschi D, Glater J, et al (2004) Advanced treatment technologies in the remediation of seleniferous drainage waters and sediments. Irrigation Drainage Syst 18: 19–42.

[4] Oman J, Dejanovic B, Tuma M (2002) Solutions to the problem of waste deposition at a coal-fired power plant. Waste Manage 22: 617–623. 10.1016/s0956-053x(01)00038-1 12214973

[5] Mehta SK, Gaur JP (2005) Use of algae for removing heavy metal ions from wastewater: Progress and prospects. Crit Rev Biotechnol 25: 113–152. 16294830 10.1080/07388550500248571

[6] Volesky B (2007) Biosorption and me. Water Res 41: 4017–4029. 17632204 10.1016/j.watres.2007.05.062

[7] Gadd GM (2009) Biosorption: Critical review of scientific rationale, environmental importance and significance for pollution treatment. J Chem Technol Biotechnol 84: 13–28.

[8] Volesky B, Holan ZR (1995) Biosorption of heavy metals. Biotechnol Prog 11: 235–250. 7619394 10.1021/bp00033a001

[9] Domozych DS, Ciancia M, Fangel JU, Mikkelsen MD, Ulvskov P, et al (2012) The cell walls of green algae: a journey through evolution and diversity. Front Plant Sci 3: 1–7. 22639667 10.3389/fpls.2012.00082 PMC3355577

[10] Davis TA, Volesky B, Mucci A (2003) A review of heavy metal biosorption by brown algae. Water Res 37: 4311–4330. 14511701 10.1016/S0043-1354(03)00293-8

[11] Latva S, Peraniemi S, Ahlgren M (2003) Study of metal-loaded activated charcoals for the separation and determination of selenium species by energy dispersive X-ray fluorescence analysis. Anal Chim Acta 478: 229–235.

[12] Bird MI, Wurster CM, Silva PHD, Bass AM, de Nys R (2011) Algal biochar: Production and properties. Bioresour Technol 102: 1886–1891. 20797850 10.1016/j.biortech.2010.07.106

[13] Roberts DA, Paul NA, de Nys R (2013) Biosorbents and methods of use. Provisional patent 27326AU1-DJH/MAR. Available: http://www.ipaustralia.com.au/applicant/james-cook-university/patents/AU2013902101/. Accessed 21 March 2014.

[14] Manceau A, Charlet L (1994) The mechanism of selenate adsorption on goethite and hydrous ferric oxide. J Colloid Interface Sci 168: 87–93.

[15] Lalvani SB (2004) Selemium removal from agricultural drainage water: Lab scale studies. Sacramento: Department of Water Resources. 86 p.

[16] Yang T, Chen M-L, Liu L-H, Wang J-H, Dasgupta PK (2012) Iron(III) Modification of *Bacillus subtilis* membranes provides record sorption capacity for arsenic and endows unusual selectivity for As(V). Environ Sci Technol 46: 2251–2256. 22296291 10.1021/es204034z

[17] Saunders RJ, Paul NA, Hu Y, de Nys R (2012) Sustainable sources of biomass for bioremediation of heavy metals in waste water derived from coal-fired power generation. PLoS ONE 7: e36470. 22590550 10.1371/journal.pone.0036470 PMC3348934

[18] Roberts D, de Nys R, Paul N (2013) The effect of CO_2_ on algal growth in industrial waste water for bioenergy and bioremediation applications. PLoS ONE 8: e81631. 24278451 10.1371/journal.pone.0081631 PMC3838398

[19] Lee HS, Suh JH, Kim B, Yoon T (2004) Effect of aluminium in two-metal biosorption by an algal biosorbent. Miner Eng 17: 487–493.

[20] Mehta SK, Tripathi BN, Gaur JP (2000). Influence of pH, temperature, culture age and cations on adsorption and uptake of Ni by *Chlorella vulgaris*. Eur J Protistol 36: 443–450. 10.1081/ese-120002837 11929076

[21] Figueira MM, Volesky B, Azarian K, Ciminelli VST (2000) Biosorption column performance with a metal mixture. Environ Sci Technol 34: 4320–4326.

[22] Figueira MM, Volesky B, Ciminelli VST (1997) Assessment of interference in biosorption of a heavy metal. Biotechnol Bioeng 54: 344–350. 18634101 10.1002/(SICI)1097-0290(19970520)54:4<344::AID-BIT7>3.0.CO;2-K

[23] Lawton RJ, de Nys R, Skinner S, Paul NA (2014) Isolation and selection of *Oedogonium* species and strains for biomass applications. PLoS ONE 9: e90223. 24603705 10.1371/journal.pone.0090223 PMC3946159

[24] Entwistle TJ, Skinner S, Lewis SH, Foard HJ (2007). Algae of Australia: Batrachospermales, Thoreales, Oedogoniales and Zygnemaceae. Canberra: CSIRO Publishing. 191 p.

[25] Jolliffe IT (2002) Principal Component Analysis. New York: Springer-Verlag. 487 p.

[26] Quinn G, Keough M (2002) Experimental design and data analysis for biologists. Cambridge: Cambridge University Press. 556 p.

[27] ANZECC (2000) Australian and New Zealand guidelines for fresh and marine water quality. Sydney: Australian Water Association. 314 p.

[28] Vieira DM, da Costa ACC, Henriques CA, Cardoso VL, de Franca FP (2007) Biosorption of lead by the brown seaweed *Sargassum filipendula* - Batch and continuous pilot studies. Electron J Biotechnol 10: 368–375.

[29] Pavasant P, Apiratikul R, Sungkhum V, Suthiparinyanont P, Wattanachira S, et al (2006) Biosorption of Cu^2+^, Cd^2+^, Pb^2+^, and Zn^2+^ using dried marine green macroalga *Caulerpa lentillifera* . Bioresour Technol 97: 2321–2329. 16330209 10.1016/j.biortech.2005.10.032

[30] Karthikeyan S, Balasubramanian R, Iyer CS (2007) Evaluation of the marine algae *Ulva fasciata* and *Sargassum* sp. for the biosorption of Cu(II) from aqueous solutions. Bioresour Technol 98: 452–455. 16530408 10.1016/j.biortech.2006.01.010

[31] Esmaeili A, Ghasemi S, Sohrabipour J (2010) Biosorption of copper from wastewater by activated carbon preparation from alga *Sargassum* sp. Nat Prod Res 24: 341–348. 20221940 10.1080/14786410903064915

[32] Schneegurt MA, Jain JC, Menicucci JA, Brown SA, Kemner KM, et al (2001) Biomass byproducts for the remediation of wastewaters contaminated with toxic metals. Environ Sci Technol 35: 3786–3791. 11783660 10.1021/es010766e

[33] Sheng PX, Ting YP, Chen JP, Hong L (2004) Sorption of lead, copper, cadmium, zinc, and nickel by marine algal biomass: Characterization of biosorptive capacity and investigation of mechanisms. J Colloid Interface Sci 275: 131–141. 15158390 10.1016/j.jcis.2004.01.036

[34] Stumm W, Morgan J (1996) Aquatic chemistry: Chemical equilibria and rates in natural waters. New York: Wiley. 1040 p.

[35] Zhang N, Lin LS, Gang DC (2008) Adsorptive selenite removal from water using iron-coated GAC adsorbents. Water Res 42: 3809–3816. 18694584 10.1016/j.watres.2008.07.025

[36] Can C, Jianlong W (2007) Correlating metal ionic characteristics with biosorption capacity using QSAR model. Chemosphere 69: 1610–1616. 17624405 10.1016/j.chemosphere.2007.05.043

[37] Zamil SS, Ahmad S, Choi MH, Park JY, Yoon SC (2009) Correlating metal ionic characteristics with biosorption capacity of *Staphylococcus saprophyticus* BMSZ711 using QICAR model. Bioresour Technol 100: 1895–1902. 19038544 10.1016/j.biortech.2008.10.014

[38] Kogej A, Pavko A (2001) Comparison of *Rhizopus nigricans* in a pelleted growth form with some other types of waste microbial biomass as biosorbents for metal ions. World J Microbiol Biotechnol 17: 677–685.

[39] Brady JM, Tobin JM (1995) Binding of hard and soft metal-ions to *Rhizopus arrhizus* biomass. Enzyme Microb Technol 17: 791–796.

[40] Tobin JM, Cooper DG, Neufeld RJ (1984) Uptake of metal ions by *Rhizopus arrhizus* biomass. Appl Environ Microbiol 47: 821–824. 16346521 10.1128/aem.47.4.821-824.1984 PMC239770

[41] Sigg L, Xue H (1994) Metal speciation: Concepts, analysis and effects. In: Bidoglio G, Stumm W, editors. Chemistry of aquatic systems: Local and global perspectives. Brussels: ECSE EAEC.pp. 153–181.

[42] Esposito A, Pagnanelli F, Veglio F (2002) pH-related equilibria models for biosorption in single metal systems. Chem Eng Sci 57: 307–313.

[43] Peterson HG, Healey FP, Wagemann R (1984) Metal toxicity to algae - A highly ph dependent phenomenon. Can J Fish Aquat Sci 41: 974–979.

[44] Pirszel J, Pawlik B, Skowronski T (1995) Cation-exchange capacity of algae and cyanobacteria: A parameter of their metal sorption abilities. J Ind Microbiol 14: 319–322.

[45] Crist RH, Martin JR, Carr D, Watson JR, Clarke HJ, et al (1994) Interaction of metals and protons with algae.4. Ion-exchange vs adsorption models and a reassessment of scatchard plots - ion-exchange rates and equilibria compared with calcium alginate. Environ Sci Technol 28: 1859–1866. 22175926 10.1021/es00060a016

[46] Garnham GW, Avery SV, Codd GA, Gadd GM (1994) Interactions of microalgae and cyanobacteria with toxic metals and radionuclides: Physiology and environmental implications. In: Dyer KR, Orth RJ, editors. Changes in fluxes in estuaries - Implications from science to management. Fredensborg: Olsen and Olsen. pp. 289–293.

[47] Schijf J, Ebling AM (2010) Investigation of the ionic strength dependence of *Ulva lactuca* acid functional group pKas by manual alkalimetric titrations. Environ Sci Technol 44: 1644–1649. 20121199 10.1021/es9029667

[48] Farrell M, Ragnott G, Krull E (2013) Difficulties in using soil-based methods to assess plant availability of potenitally toxic elements in biochars and their feedstocks. J Hazard Mat 250–251: 29–36. 10.1016/j.jhazmat.2013.01.073 23454453

