# Supplementary material for: Bioremediation of a Complex Industrial Effluent by Biosorbents Derived from Freshwater Macroalgae
Source: PLoS One. 2014 Jun 11;9(6):e94706. doi: 10.1371/journal.pone.0094706 (PMC4053327; doi:10.1371/journal.pone.0094706)
Supplement: Figure S1 — Principal Components Analysis of solution concentration for 12 ANZECC elements. (A) PCA and (B) factor loadings for 12 elements include all biosorbent, time periods (excluding t0) and pH conditions, grouped by biosorbent. Vectors (factor loadings) indicate the direction and magnitude of correlation between a specific element and the biosorbent which resulted in the lowest respective concentration. (DOCX) [file pone.0094706.s001.docx]

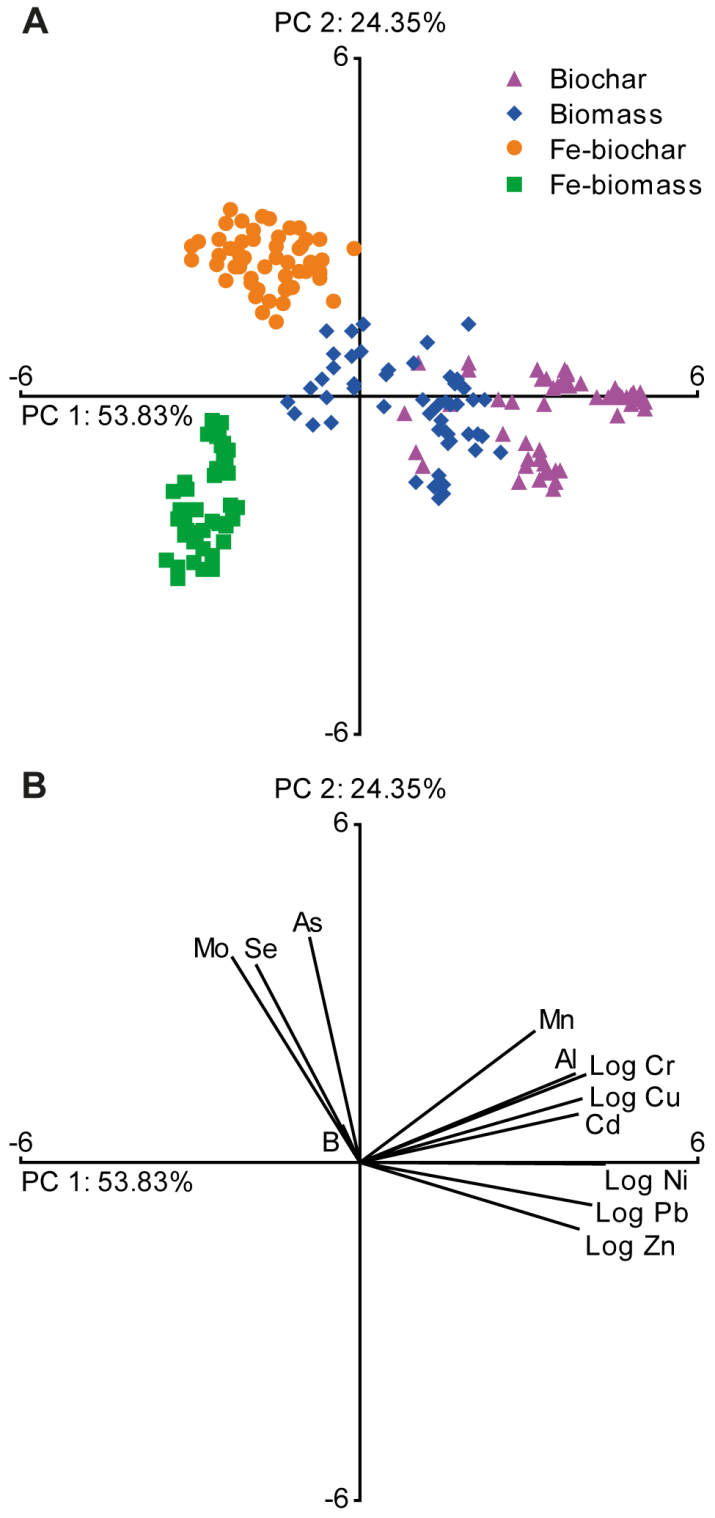


**Figure S1.** **Principal Components Analysis of solution concentration for 12 ANZECC elements.** (A) PCA and (B) factor loadings for 12 elements include all biosorbent, time periods (excluding t0) and pH conditions, grouped by biosorbent. Vectors (factor loadings) indicate the direction and magnitude of correlation between a specific element and the biosorbent which resulted in the lowest respective concentration.
