# Supplementary material for: Bioremediation of a Complex Industrial Effluent by Biosorbents Derived from Freshwater Macroalgae
Source: PLoS One. 2014 Jun 11;9(6):e94706. doi: 10.1371/journal.pone.0094706 (PMC4053327; doi:10.1371/journal.pone.0094706)
Supplement: Figure S2 — The total respective biosorption of metals (Al, Cd, Cr, Cu, Pb, Mn, Ni, and Zn) by (a) Fe-biomass and (b) Fe-biochar and total respective biosorption of metalloids (As, B, Mo and Se) by (c) biochar and (d) biomass. Initial pH of 2.5, 4 and un-manipulated (7.1) are shown by solid, dashed and dotted lines, respectively. Error bars show standard errors. (DOCX) [file pone.0094706.s002.docx]

**Figure S2:** The total respective biosorption of metals (Al, Cd, Cr, Cu, Pb, Mn, Ni, and Zn) by (a) Fe-biomass and (b) Fe-biochar and total respective biosorption of metalloids (As, B, Mo and Se) by (c) biochar and (d) biomass. Initial pH of 2.5, 4 and un-manipulated (7.1) are shown by solid, dashed and dotted lines, respectively. Error bars show standard errors.
