# Supplementary material for: Bioremediation of a Complex Industrial Effluent by Biosorbents Derived from Freshwater Macroalgae
Source: PLoS One. 2014 Jun 11;9(6):e94706. doi: 10.1371/journal.pone.0094706 (PMC4053327; doi:10.1371/journal.pone.0094706)
Supplement: Figure S3 — The biosorption of ANZECC metals (Pb, Cr, Cu, and Mn) when exposed to biochar and biomass. Initial pH of 2.5, 4 and un-manipulated (7.1) are shown by solid, dashed and dotted lines, respectively. Error bars show standard errors. Horizontal dashed line indicates the respective ANZECC trigger concentration for each element. (DOCX) [file pone.0094706.s003.docx]

**Figure S3:** The biosorption of ANZECC metals (Pb, Cr, Cu, and Mn) when exposed to biochar and biomass. Initial pH of 2.5, 4 and un-manipulated (7.1) are shown by solid, dashed and dotted lines, respectively. Error bars show standard errors. Horizontal dashed line indicates the respective ANZECC trigger concentration for each element.
