# Supplementary material for: Bioremediation of a Complex Industrial Effluent by Biosorbents Derived from Freshwater Macroalgae
Source: PLoS One. 2014 Jun 11;9(6):e94706. doi: 10.1371/journal.pone.0094706 (PMC4053327; doi:10.1371/journal.pone.0094706)
Supplement: Table S1 — Three factor factorial Analysis of Variance tests run on each of the 12 ANZECC elements. Factorial analysis of variance tests were run on elemental concentration under the factors of Biosorbent, pH (Fixed) and Time (Random). Type III sum of squares was used. All tests met the assumption of homogeneity of variance, normality of residuals and independence. Transformation of the data were required for some elements, the transformation applied is listed next to the title. Factors in bold indicate significance under alpha of 0.05. (DOCX) [file pone.0094706.s004.docx]

**Table S1:** Three factor factorial Analysis of Variance tests run on each of the 12 ANZECC elements. Factorial analysis of variance tests were run on elemental concentration under the factors of Biosorbent, pH (Fixed) and Time (Random). Type III sum of squares was used. All tests met the assumption of homogeneity of variance, normality of residuals and independence. Transformation of the data were required for some elements, the transformation applied is listed next to the title. Factors in bold indicate significance under alpha of 0.05.

| Factor | Effect | DF | SS | MS | F | p |
| --- | --- | --- | --- | --- | --- | --- |
| Aluminium | | | | | | |
| **Biosorbent** | **Fixed** | **3** | **2.9E+07** | **9.6E+06** | **159** | **<0.001** |
| **pH** | **Fixed** | **2** | **4.2E+05** | **2.1E+05** | **56.9** | **<0.001** |
| Time | Random | 5 | 5.8E+05 | 1.2E+05 | 2.12 | 0.132 |
| **Biosorbent*pH** | **Fixed** | **6** | **3.7E+05** | **6.1E+04** | **6.75** | **<0.001** |
| **Biosorbent*Time** | **Random** | **15** | **9.1E+05** | **6.1E+04** | **6.67** | **<0.001** |
| pH*Time | Random | 10 | 3.7E+04 | 3.7E+03 | 0.41 | 0.933 |
| Biosorbent*pH*Time | Random | 30 | 2.7E+05 | 9.1E+03 | 0.27 | 1.000 |
| Error |  | 144 | 4.9E+06 | 3.4E+04 |  |  |
| Arsenic [Cuberoot] | | | | | | |
| **Biosorbent** | **Fixed** | **3** | **39.3** | **13.1** | **81.0** | **<0.001** |
| **pH** | **Fixed** | **2** | **0.09** | **0.04** | **17.9** | **<0.001** |
| Time | Random | 5 | 1.56 | 0.31 | 1.93 | 0.149 |
| **Biosorbent*pH** | **Fixed** | **6** | **0.34** | **0.06** | **20.6** | **<0.001** |
| **Biosorbent*Time** | **Random** | **15** | **2.43** | **0.16** | **58.7** | **<0.001** |
| pH*Time | Random | 10 | 0.02 | 0.00 | 0.89 | 0.555 |
| Biosorbent*pH*Time | Random | 30 | 0.08 | 0.00 | 0.05 | 1.000 |
| Error |  | 144 | 7.73 | 0.05 |  |  |

| Factor | Effect | | DF | | SS | | MS | | F | | p | |
| --- | --- | --- | --- | --- | --- | --- | --- | --- | --- | --- | --- | --- |
| Boron | | | | | | | | | | | | |
| **Biosorbent** | | **Fixed** | | **3** | | **5.5E+06** | | **1.8E+06** | | **3.81** | | **0.033** |
| pH | | Fixed | | 2 | | 4.7E+05 | | 2.4E+05 | | 2.18 | | 0.164 |
| **Time** | | **Random** | | **5** | | **2.2E+07** | | **4.3E+06** | | **10.8** | | **0.001** |
| Biosorbent*pH | | Fixed | | 6 | | 2.0E+06 | | 3.3E+05 | | 1.78 | | 0.138 |
| **Biosorbent*Time** | | **Random** | | **15** | | **7.2E+06** | | **4.8E+05** | | **2.59** | | **0.013** |
| pH*Time | | Random | | 10 | | 1.1E+06 | | 1.1E+05 | | 0.58 | | 0.815 |
| Biosorbent*pH*Time | | Random | | 30 | | 5.6E+06 | | 1.9E+05 | | 0.08 | | 1.000 |
| Error | |  | | 144 | | 3.3E+08 | | 2.3E+06 | |  | |  |
| Cadmium | | | | | | | | | | | | |
| **Biosorbent** | | **Fixed** | | **3** | | **33.3** | | **11.1** | | **125** | | **<0.001** |
| **pH** | | **Fixed** | | **2** | | **4.97** | | **2.49** | | **24.2** | | **<0.001** |
| Time | | Random | | 5 | | 0.65 | | 0.13 | | 0.94 | | 0.491 |
| **Biosorbent*pH** | | **Fixed** | | **6** | | **5.47** | | **0.91** | | **17.0** | | **<0.001** |
| Biosorbent*Time | | Random | | 15 | | 1.33 | | 0.09 | | 1.66 | | 0.116 |
| pH*Time | | Random | | 10 | | 1.03 | | 0.10 | | 1.92 | | 0.082 |
| Biosorbent*pH*Time | | Random | | 30 | | 1.61 | | 0.05 | | 1.19 | | 0.243 |
| Error | |  | | 144 | | 6.47 | | 0.04 | |  | |  |
| Chromium [Squareroot] | | | | | | | | | | | | |
| **Biosorbent** | | **Fixed** | | **3** | | **4815** | | **1605** | | **418** | | **<0.001** |
| **pH** | | **Fixed** | | **2** | | **3.20** | | **1.60** | | **7.03** | | **0.012** |
| **Time** | | **Random** | | **5** | | **104** | | **20.8** | | **5.37** | | **0.005** |
| **Biosorbent*pH** | | **Fixed** | | **6** | | **6.06** | | **1.01** | | **5.24** | | **0.001** |
| **Biosorbent*Time** | | **Random** | | **15** | | **57.6** | | **3.84** | | **19.9** | | **<0.001** |
| pH*Time | | Random | | 10 | | 2.28 | | 0.23 | | 1.18 | | 0.341 |
| Biosorbent*pH*Time | | Random | | 30 | | 5.78 | | 0.19 | | 0.13 | | 1.000 |
| Error | |  | | 144 | | 221 | | 1.53 | |  | |  |

| Factor | Effect | | DF | | SS | | MS | | F | | p | |
| --- | --- | --- | --- | --- | --- | --- | --- | --- | --- | --- | --- | --- |
| Copper [Cuberoot] | | | | | | | | | | | | |
| **Biosorbent** | | **Fixed** | | **3** | | **570** | | **190** | | **816** | | **<0.001** |
| **pH** | | **Fixed** | | **2** | | **0.08** | | **0.04** | | **4.66** | | **0.037** |
| Time | | Random | | 5 | | 2.39 | | 0.48 | | 2.06 | | 0.129 |
| **Biosorbent*pH** | | **Fixed** | | **6** | | **1.61** | | **0.27** | | **32.4** | | **<0.001** |
| **Biosorbent*Time** | | **Random** | | **15** | | **3.49** | | **0.23** | | **28.1** | | **<0.001** |
| pH*Time | | Random | | 10 | | 0.08 | | 0.01 | | 0.97 | | 0.488 |
| Biosorbent*pH*Time | | Random | | 30 | | 0.25 | | 0.01 | | 0.06 | | 1.000 |
| Error | |  | | 144 | | 21.6 | | 0.15 | |  | |  |
| Lead [Squareroot] | | | | | | | | | | | | |
| **Biosorbent** | | **Fixed** | | **3** | | **167** | | **55.7** | | **129** | | **<0.001** |
| **pH** | | **Fixed** | | **2** | | **0.87** | | **0.44** | | **9.79** | | **0.004** |
| Time | | Random | | 5 | | 2.01 | | 0.40 | | 0.90 | | 0.504 |
| Biosorbent*pH | | Fixed | | 6 | | 0.40 | | 0.07 | | 2.36 | | 0.055 |
| **Biosorbent*Time** | | **Random** | | **15** | | **6.46** | | **0.43** | | **15.2** | | **<0.001** |
| pH*Time | | Random | | 10 | | 0.45 | | 0.04 | | 1.58 | | 0.162 |
| Biosorbent*pH*Time | | Random | | 30 | | 0.85 | | 0.03 | | 0.87 | | 0.663 |
| Error | |  | | 144 | | 4.68 | | 0.03 | |  | |  |
| Manganese [Log] | | | | | | | | | | | | |
| **Biosorbent** | | **Fixed** | | **3** | | **225** | | **75.0** | | **330** | | **<0.001** |
| **pH** | | **Fixed** | | **2** | | **28.6** | | **14.3** | | **127** | | **<0.001** |
| Time | | Random | | 5 | | 3.50 | | 0.70 | | 2.49 | | 0.074 |
| **Biosorbent*pH** | | **Fixed** | | **6** | | **52.3** | | **8.72** | | **147** | | **<0.001** |
| **Biosorbent*Time** | | **Random** | | **15** | | **3.41** | | **0.23** | | **3.84** | | **<0.001** |
| pH*Time | | Random | | 10 | | 1.13 | | 0.11 | | 1.90 | | 0.085 |
| Biosorbent*pH*Time | | Random | | 30 | | 1.78 | | 0.06 | | 0.88 | | 0.653 |
| Error | |  | | 144 | | 9.73 | | 0.07 | |  | |  |

| Factor | Effect | | DF | | SS | | MS | | F | | p | |
| --- | --- | --- | --- | --- | --- | --- | --- | --- | --- | --- | --- | --- |
| Molybdenum [Cuberoot] | | | | | | | | | | | |  |
| **Biosorbent** | | **Fixed** | | **3** | | **984** | | **328** | | **47.1** | **<0.001** |  |
| **pH** | | **Fixed** | | **2** | | **14.0** | | **6.98** | | **113** | **<0.001** |  |
| Time | | Random | | 5 | | 52.3 | | 10.5 | | 1.52 | 0.244 |  |
| **Biosorbent*pH** | | **Fixed** | | **6** | | **45.1** | | **7.52** | | **54.0** | **<0.001** |  |
| **Biosorbent*Time** | | **Random** | | **15** | | **104** | | **6.96** | | **50.0** | **<0.001** |  |
| pH*Time | | Random | | 10 | | 0.62 | | 0.06 | | 0.45 | 0.911 |  |
| Biosorbent*pH*Time | | Random | | 30 | | 4.17 | | 0.14 | | 0.61 | 0.946 |  |
| Error | |  | | 144 | | 33.1 | | 0.23 | |  |  |  |
| Nickel [Log] | | | | | | | | | | | |  |
| **Biosorbent** | | **Fixed** | | **3** | | **355** | | **118** | | **183** | **<0.001** |  |
| **pH** | | **Fixed** | | **2** | | **2.04** | | **1.02** | | **10.5** | **0.003** |  |
| Time | | Random | | 5 | | 1.90 | | 0.38 | | 0.57 | 0.722 |  |
| **Biosorbent*pH** | | **Fixed** | | **6** | | **1.60** | | **0.27** | | **3.58** | **0.008** |  |
| **Biosorbent*Time** | | **Random** | | **15** | | **9.68** | | **0.65** | | **8.68** | **<0.001** |  |
| pH*Time | | Random | | 10 | | 0.97 | | 0.10 | | 1.31 | 0.271 |  |
| Biosorbent*pH*Time | | Random | | 30 | | 2.23 | | 0.07 | | 0.90 | 0.621 |  |
| Error | |  | | 144 | | 11.9 | | 0.08 | |  |  |  |
| Selenium | | | | | | | | | | | |  |
| **Biosorbent** | | **Fixed** | | **3** | | **86006** | | **28669** | | **49.4** | **<0.001** |  |
| **pH** | | **Fixed** | | **2** | | **1556** | | **778** | | **13.7** | **0.001** |  |
| Time | | Random | | 5 | | 7719 | | 1544 | | 2.73 | 0.064 |  |
| **Biosorbent*pH** | | **Fixed** | | **6** | | **4729** | | **788** | | **10.9** | **<0.001** |  |
| **Biosorbent*Time** | | **Random** | | **15** | | **8709** | | **581** | | **8.05** | **<0.001** |  |
| pH*Time | | Random | | 10 | | 567 | | 57 | | 0.79 | 0.642 |  |
| **Biosorbent*pH*Time** | | **Random** | | **30** | | **2164** | | **72** | | **1.91** | **0.006** |  |
| Error | |  | | 144 | | 5447 | | 38 | |  |  |  |

| Factor | Effect | | DF | | SS | | MS | | F | | p | |
| --- | --- | --- | --- | --- | --- | --- | --- | --- | --- | --- | --- | --- |
| Zinc [Cuberoot] | | | | | | | | | | | | |
| **Biosorbent** | | **Fixed** | | **3** | | **849** | | **283** | | **30.9** | | **<0.001** |
| **pH** | | **Fixed** | | **2** | | **157** | | **78.6** | | **96.8** | | **<0.001** |
| Time | | Random | | 5 | | 7.29 | | 1.46 | | 0.17 | | 0.970 |
| **Biosorbent*pH** | | **Fixed** | | **6** | | **152** | | **25.4** | | **19.4** | | **<0.001** |
| **Biosorbent*Time** | | **Random** | | **15** | | **137** | | **9.16** | | **7.00** | | **<0.001** |
| pH*Time | | Random | | 10 | | 8.12 | | 0.81 | | 0.62 | | 0.784 |
| **Biosorbent*pH*Time** | | **Random** | | **30** | | **39.2** | | **1.31** | | **4.03** | | **<0.001** |
| Error | |  | | 144 | | 46.8 | | 0.33 | |  | |  |
